# The therapeutic value of the SphK1-targeting microRNA-3677 in human osteosarcoma cells

**DOI:** 10.18632/aging.102961

**Published:** 2020-03-23

**Authors:** Chen Yao, Jian-Wei Ruan, Yun-Rong Zhu, Fei Liu, Hui-ming Wu, Yan Zhang, Qing Jiang

**Affiliations:** 1 Department of Orthopedics, Nanjing Drum Tower Hospital of Nanjing Medical University, Nanjing, China; 2 Department of Orthopedics, Affiliated Hospital of Nanjing University of TCM, Jiangsu Province Hospital of TCM, Nanjing, China; 3 Department of Orthopedics, Taizhou Municipal Hospital, Taizhou, China; 4 Department of Orthopedics, The Affiliated Jiangyin Hospital of Medical College of Southeast University, Jiangyin, China; 5 Department of Orthopedics, Children’s Hospital of Nanjing Medical University, Nanjing, China; 6 Department of Radiotherapy and Oncology, Affiliated Kunshan Hospital of Jiangsu University, Kunshan, China

**Keywords:** osteosarcoma, miRNA-3677, SphK1, cell apoptosis, signaling

## Abstract

Sphingosine kinase 1 (SphK1) is a potential therapeutic target for human osteosarcoma (OS). SphK1-targeting microRNAs (miRNAs) could have important therapeutic value for OS. We discovered that micorRNA-3677 (miR-3677) is a SphK1-targeting miRNA, inhibiting OS cell progression. The results of RNA-Pull down assay confirmed direct binding between biotinylated-miR-3677 and *SphK1* mRNA in primary human OS cells. In established and primary human OS cells forced overexpression of miR-3677, by a lentiviral construct, decreased SphK1 3’-UTR (untranslated region) activity and downregulated SphK1 expression. Both were however enhanced with miR-3677 inhibition in OS cells. Function studies demonstrated that OS cell growth, proliferation and migration were inhibited with miR-3677 overexpression, but augmented with miR-3677 inhibition. MiR-3677 overexpression-induced anti-OS cell activity was reversed with re-expression of the 3’-UTR-depleted *SphK1*. Additionally, in SphK1 knockout OS cells (by CRISPR/Cas9 strategy), altering miR-3677 expression failed to further alter cell functions. Finally, we show that miR-3677 expression was significantly downregulated in primary human OS tissues, correlating with *SphK1* mRNA upregulation. We conclude that targeting SphK1 by miR-3677 inhibits human OS cell progression.

## INTRODUCTION

Osteosarcoma (OS) is a common malignant bone tumor [1, 2]. Each year it is estimated that over three million new cases of OS will be diagnosed, mostly in children and adolescents [1, 2]. OS survival has been significantly improved over the past decades, owing to progress in the early disease diagnosis techniques and latest developments in molecularly-targeted and/or combination therapies [1, 2]. For the recurrent and metastatic OS, the current clinical therapies are limited [1, 2]. Therefore, it is important to explore novel and reliable molecular targets for OS efficient therapy [1, 2]. It is also the research focus of our group [3–5].

Sphingosine kinase (SphK) family proteins, including SphK1 and SphK2, catalyze sphingosine phosphorylation to form sphingosine-1-phosphate (S1P) [6], the latter is a key lipid mediator with intracellular and extracellular functions [7]. SphK1 regulates the balance between lipid mediators, including ceramide, sphingosine, and S1P. SphK1 inhibition, silencing or loss-of-function mutation will lead to S1P depletion and ceramide accumulation, causing significant cell death and apoptosis [7]. However, in many types of human cancers, SphK1 overexpression and/or over-activation would promote cancer cell growth and proliferation [7]. Our previous studies have shown that SphK1 is overexpressed in human OS, representing as an important therapeutic target [5].

MicroRNAs (miRNAs) are a large family of conserved small non-coding RNAs, ranging from 21-25-nucleotide in length [8, 9]. MiRNAs can regulate gene expression at the translational and post-transcriptional levels [8, 9]. MiRNAs directly bind to the 3’ untranslated region (3’-UTR) of the complementary mRNAs, thereby inhibiting mRNA translation and/or inducing degradation of targeted mRNAs [8, 9]. Dysregulation of miRNAs is commonly detected in human OS [10–12], associated with OS tumorigenesis, pathogenesis and progression [8, 9].

One promising strategy to inhibit SphK1-induced cancer progression is to express SphK1-targeting miRNAs. Zhou et al., demonstrated that miR-124 inhibited OS cell proliferation and invasion via directly targeting SphK1 [13]. Lu et al., showed that miRNA-101 silenced SphK1 to inhibit colorectal cancer cell progression [14]. Similarly, miR-506 inhibited liver cancer angiogenesis through silencing SphK1 [15]. The present study discovered micorRNA-3677 (miR-3677), as a SphK1-targeting miRNA, efficiently inhibits OS cell progression by targeting and silencing SphK1.

## RESULTS

### MiR-3677 targets and downregulates SphK1 in human OS cells

**Figure 1:**
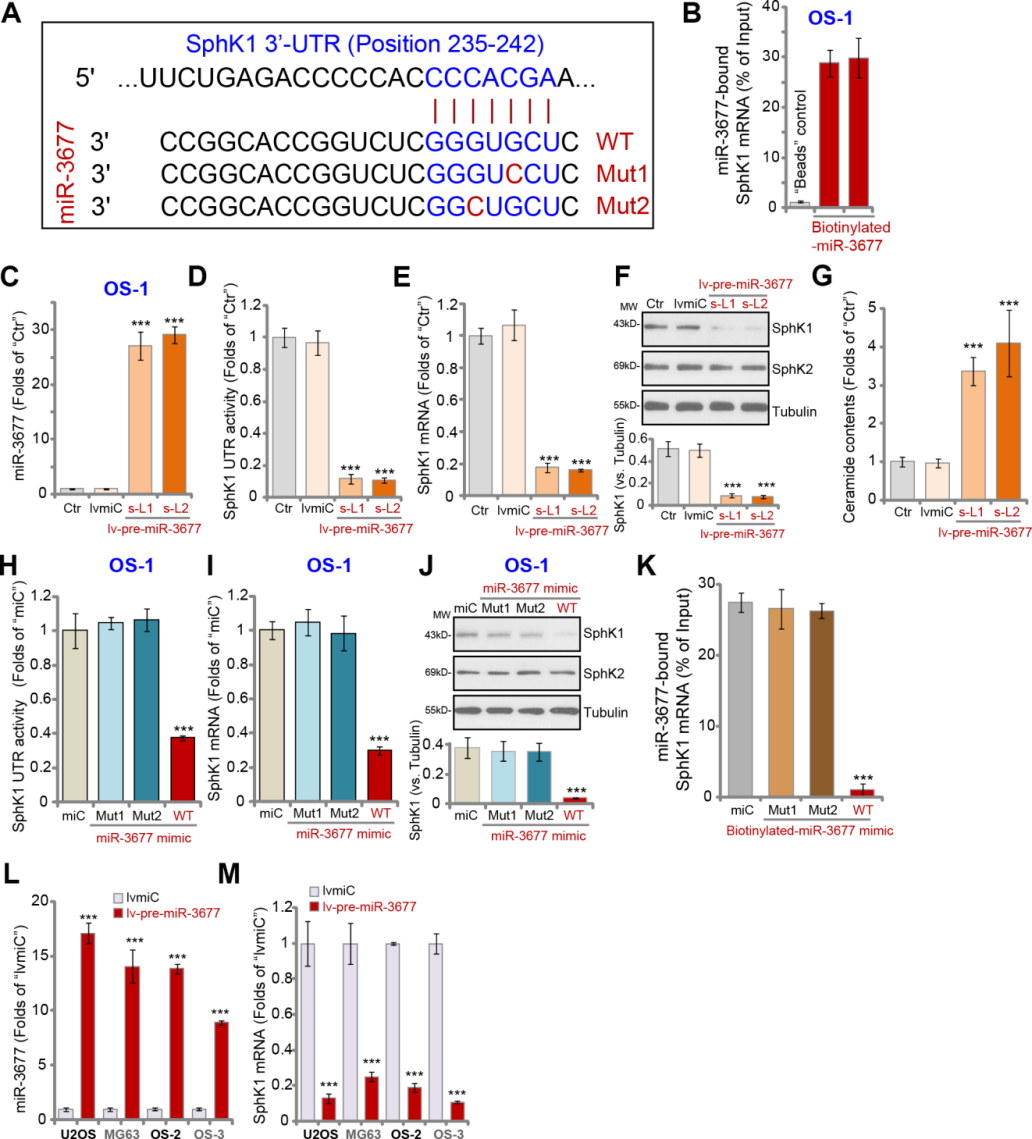
**MiR-3677 targets and downregulates SphK1 in human OS cells.** MiR-3677 (-3p) putatively targets the 3’-UTR (untranslated region) of human *SphK1* (at position 235-242) (**A**). RNA-Pull down assay results in primary human OS-1 cells demonstrated the direct association between biotinylated-miR-3677 and *SphK1* mRNA (**B**). In parental control OS-1 cells (“Ctr”), stable OS-1 cells with pre-miR-3677-expressing lentivirus (“lv-pre-miR-3677”, s-L1/s-L2, two lines) or with the lentiviral non-sense control miRNA (“lvmiC”) construct, expression of mature miR-3677 (-3p, **C**), *SphK1* mRNA (**E**) and listed proteins (**F**) were tested by qPCR and Western blotting assays, with the relative SphK1 3’-UTR activity (**D**) and ceramide contents (**G**) tested as well. OS-1 cells were transfected with 500 nM of non-sense microRNA control (“miC”), the wild-type (“WT”) or the mutant miR-3677 (-3p) mimics (sequences listed in A, “Mut1/2”), with SphK1 3’-UTR activity (**H**) and *SphK1* mRNA (**I**)/protein (**J**) expression tested after 48h. Furthermore, *SphK1* mRNA directly binds to biotinylated-WT miR-3677, but not to the mutants (“Mut1/2”, -biotinylated) in OS-1 cells (**K**). U2OS and MG63 cells as well as primary human OS cells (OS-2 and OS-3) were infected with lv-pre-miR-3677 or lvmiC, after 48h expression of mature miR-3677 (-3p, **L**) and *SphK1* mRNA (**M**) was tested. Data were presented as mean ± SD (n=5), and results were normalized. ***P< 0.001 vs. “lvmiC”/“miC” cells. Experiments in this figure were repeated five times with similar results obtained.

To explore SphK1-targeting miRNAs, the miRNA database, TargetScan (V7.2, http://targetscan.org, V7.2) [16] was consulted. A number of miRNAs potentially targeting 3’-UTR of *SphK1* were identified, then were further verified by other databases, including miRbase and miRDB. The bioinformatics studies discovered that miR-3677 (-3p) putatively targets 3’-UTR of *SphK1* (at position of 235-242) (Figure 1A). The context^++^ score for miR-3677-SphK1 3’-UTR binding is -0.78, and the score percentage is 99% (TargetScan V7.2, Figure 1A). The scores indicated a high percentage of binding between the two [16]. The RNA-Pull down assay results, Figure 1B, demonstrated that the biotinylated-miR-3677 binds to *SphK1 mRNA* in OS-1 primary human OS cells. As expected, in the negatively control, streptavidin-coated magnetic beads (“Beads”), did not bind to *SphK1 mRNA* (Figure 1B).

To test whether miR-3677 could affect SphK1 expression, OS-1 cells were infected with pre-miR-3677-expressing lentivirus (lv-pre-miR-3677). Subject to selection by puromycin two stable cell lines, “s-L1” and “s-L2”, were established. qPCR results, Figure 1C, demonstrated that the mature miR-3677 (-3p) levels increased over 25 folds in lv-pre-miR-3677-expressing OS-1 cells. Conversely, the 3’-UTR activity of *SphK1* decreased over 80% in miR-3677-overexpressed OS-1 cells (Figure 1D). *SphK1* mRNA expression decreased as well (Figure 1E). Further, miR-3677 overexpression downregulated SphK1 protein in OS-1 cells (Figure 1F), without affecting SphK2 expression (Figure 1F). With SphK1 downregulation, the cellular ceramide contents were significantly increased in miR-3677-overexpressed OS-1 cells (Figure 1G). The lentiviral construct with non-sense control miRNA (“lvmiC”) did not alter expression of miR-3677 and SphK1 in OS-1 cells (Figure 1C–1G).

To further confirm that miR-3677 specifically targets and negatively regulates SphK1, we synthesized both wild type (WT) and mutant (Mut) miR-3677 (-3p) mimics. The two mutant mimics, “Mut1” and “Mut2”, contained mutations at their binding sites to *SphK1*’s 3’-UTR (see sequences in Figure 1A). As demonstrated, in OS-1 cells transfection of the WT miR-3677 mimic decreased *SphK1* 3’-UTR activity (Figure 1H) as well as *SphK1* mRNA (Figure 1I) and protein (Figure 1J) expression. The two mutants were completely ineffective (Figure 1H–1J). Significantly, in human OS-1 cells *SphK1* mRNA failed to bind to the mutant miR-3677 (“Mut1/2”, -biotinylated), but was enriched in biotinylated WT-miR-3677 (Figure 1K).

The miR-3677’s activity in other OS cells was studied next. In U2OS/MG63 cells and primary human OS cells (OS-2 and OS-3, derived from two other patients), infection of lv-pre-miR-3677 for 48h led to upregulation of mature miR-3677 (Figure 1L), leading to *SphK1* mRNA reduction (Figure 1M). These results show that miR-3677 targets and silences SphK1 in human OS cells.

### Ectopic miR-3677 overexpression inhibits OS cell progression *in vitro*

**Figure 2:**
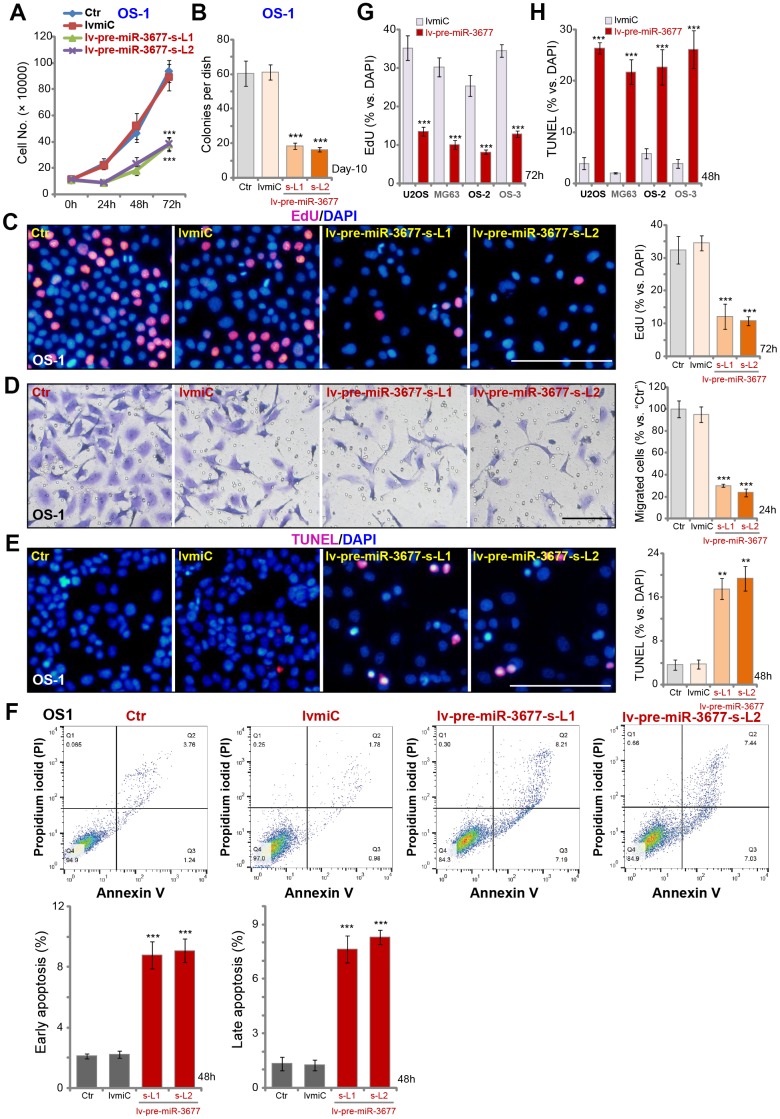
**Ectopic overexpression inhibits OS cell progression *in vitro.*** Sable OS-1 cells with the pre-miR-3677-expressing lentivirus (“lv-pre-miR-3677”, s-L1/s-L2, two lines) or with non-sense control miRNA (“lvmiC”), as well as the parental control OS-1 cells (“Ctr”), were cultured, with cell growth curve shown in (**A**); Cell colony formation (**B**), proliferation (EdU incorporation, **C**) and migration (“Transwell” assay, **D**) were tested by mentioned assays, with cell apoptosis examined by TUNEL staining (**E**) and Annexin V FACS (**F**) assays. U2OS cells and MG63 cells as well as primary human OS cells (OS-2 and OS-3) were infected with lv-pre-miR-3677 or lvmiC for indicated time periods, cell proliferation and apoptosis were tested by EdU incorporation (**G**) and TUNEL staining (**H**), respectively. For *in vitro* cell functional assays, the exact same number of viable cells with different genetic modifications were initially plated into each well/dish (at 0h/Day-0, same for all figures). Data were presented as mean ± SD (n=5), and results were normalized. ***P< 0.001 vs. “lvmiC”/“miC” cells. Experiments in this figure were repeated five times with similar results obtained. Bar=100 μm (**C**–**E**).

We have previously shown that SphK1 overexpression in OS cells is important for cell progression [5]. As shown, stable OS-1 cells with lv-pre-miR-3677 grew slower than the control cells (Figure 2A). Furthermore, OS-1 cell colony formation was inhibited over 60% with miR-3677 overexpression (Figure 2B). Results in Figure 2C demonstrated that lv-pre-miR-3677 inhibited EdU incorporation in OS-1 cells. Performing “Transwell” assays, we demonstrated that ectopic miR-3677 overexpression suppressed OS-1 cell migration by about 60-70% (Figure 2D). These results demonstrated that ectopic overexpression of miR-3677 inhibited OS-1 cell growth, proliferation and migration.

SphK1 silencing will lead to pro-apoptotic ceramides production, inducing cell cycle arrest and cell apoptosis [7, 17]. Results in Figure 1G confirmed that the ceramide contents were increased in miR-3677-overexpressed OS-1 cells. As demonstrated, Figure 2E, that nuclear TUNEL staining was increased in lv-pre-miR-3677-expressing OS-1 cells. Annexin V FACS studies confirmed that forced miR-3677 overexpression increased the percentage of both early apoptotic cells (Annexin V^+/+^ and PI^-/-^) and late apoptotic cells (Annexin V^+/+^ and PI^+/+^) (Figure 2F). In U2OS/MG63 cells and primary human OS cells (OS-2 and OS-3), lv-pre-miR-3677 similarly inhibited cell proliferation (EdU incorporation, Figure 2G), whereas inducing apoptosis activation (increased nuclear TUNEL ratio, Figure 2H). Collectively, these results show that miR-3677 overexpression inhibited OS cell progression *in vitro*.

### Forced miR-3677 inhibition increases SphK1 expression, promoting OS cell progression *in vitro*

**Figure 3:**
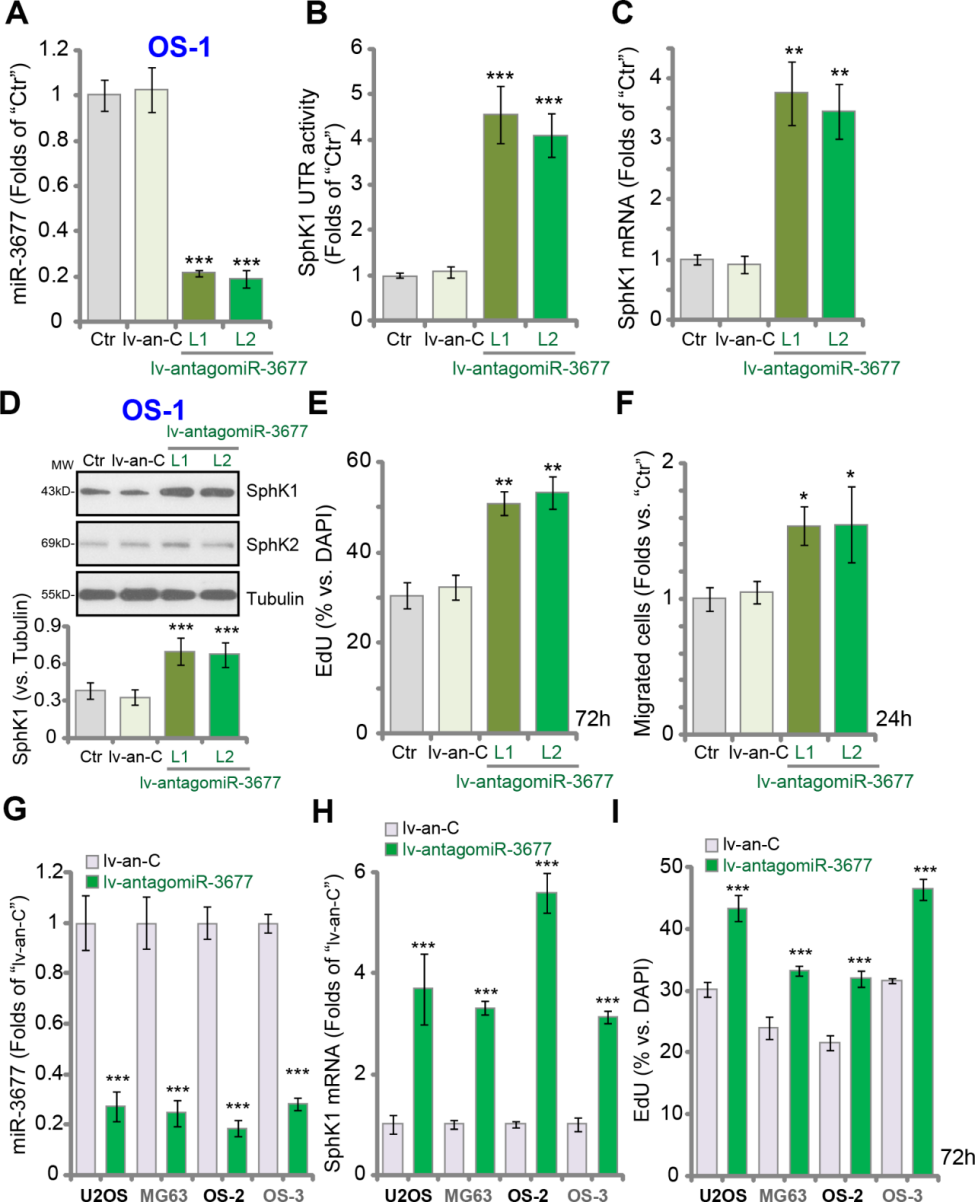
**Forced inhibition increases SphK1 expression, promoting OS cell progression *in vitro.*** Expression of listed genes in parental control OS-1 cells (“Ctr”), OS-1 cells with pre-miR-3677 anti-sense lentivirus (“lv-antagomiR-3677”, L1/ L2, two lines) or non-sense anti-sense construct (“lv-an-C”), was tested by qPCR (**A** and **C**) and Western blotting (**D**) assays, with the relative *SphK1* 3’-UTR activity examined (**B**); Cell proliferation and migration were tested by EdU incorporation (**E**) and “Transwell” (**F**) assays, respectively. The listed OS cells were infected with lv-antagomiR-3677 or lv-an-C for 48h, expression of mature miR-3677 (-3p, **G**) and *SphK1* mRNA (**H**) was tested, with cell proliferation examined by EdU incorporation assays (**I**). Expression of listed proteins was quantified, normalized to the loading control Tubulin (**G**). Data were presented as mean ± SD (n=5), and results were normalized. ***P< 0.001 vs. “lv-an-C” cells. Experiments in this figure were repeated five times with similar results obtained.

To inhibit miR-3677, the lentivirus encoding the anti-sense sequence of pre-miR-3677 (lv-antagomiR-3677) was transduced to OS-1 cells. Two stable cell lines, lv-antagomiR-3677-L1/L2, were established, and mature miR-3677 expression decreased over 70-80% (*vs.* control cells) (Figure 3A). In OS-1 cells miR-3677 inhibition, by lv-antagomiR-3677, resulted in 4-5-fold increase of SphK1’s 3’-UTR activity (Figure 3B). As a result *SphK1* mRNA (Figure 3C) and protein (Figure 3D) expression were elevated, and SphK2 expression was unchanged (Figure 3D). Functional studies demonstrated that miR-3677 inhibition in OS-1 cells promoted cell proliferation (EdU incorporation, Figure 3E) and migration (“Transwell” assay, Figure 3F). Similar results were obtained in U2OS-MG63 cells and other primary human OS cells (OS-2 and OS-3). Lv-antagomiR-3677 led to miR-3677 downregulation (Figure 3G), *SphK1* mRNA elevation (Figure 3H) and enhanced cell proliferation (Figure 3I) in OS cells. Collectively, these results show that forced miR-3677 inhibition increased SphK1 expression, promoting OS cell progression *in vitro*.

### MiR-3677 overexpression inhibits OS cell progression by targeting SphK1

**Figure 4:**
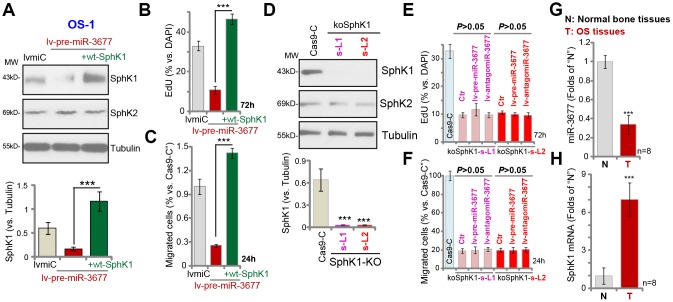
**MiR-3677 overexpression inhibits OS cell progression by targeting SphK1.** Stable OS-1 cells with pre-miR-3677-expressing lentivirus (“lv-pre-miR-3677”, s-L1) were further transfected with lentiviral SphK1-expresing construct (“+wt-SphK1”), control cells were transduced with the lentiviral construct with control miRNA (“lvmiC”), expression of listed proteins was shown (**A**); Cells were further cultured, cell proliferation and migration were tested by EdU incorporation (**B**) and “Transwell” assay (**C**), respectively; and results were quantified (**B** and **C**). Expression of listed proteins in stable OS-1 cells with the CRISPR-Cas9-SphK1-KO-GFP construct (“koSphK1-s-L1/koSphK1-s-L2”, two lines) or control construct (“Cas-9-C”) was shown (**D**). The koSphK1 cells were further infected with lv-antagomiR-3677 or lv-pre-miR-3677 for 48h, with cell proliferation (**E**) and migration (**F**) tested; and results were quantified. Expression of miR-3677 (**G**) and SphK1 mRNA (**H**) in eight (n=8) different human OS tissues (“T”) and surrounding normal bone tissues (“N”) was tested. Data were presented as mean ± SD, and results were normalized. ***P< 0.001 (**A**–**C**). ***P< 0.001 vs. “Cas-9-C” cells (**D**). ***P< 0.001 vs. “N” tissues (**G** and **H**). Experiments in this figure were repeated five times with similar results obtained.

Next, experiments were performed to test whether SphK1 silencing is the primary cause of miR-3677 overexpression-induced anti-OS cell activity. To the lv-pre-miR-3677-s-L1 OS-1 cells (see Figures 1 and 2), a lentiviral *SphK1*-expresing construct (“wt-SphK1”, without 3’-UTR) was transduced, thus restoring SphK1 expression (Figure 4A). SphK1 expression in the wt-SphK1 cells was even higher than that in the control lvmiC cells (Figure 4A). As demonstrated, lv-pre-miR-3677-induced inhibitions on cell proliferation (EdU incorporation, Figure 4B) and migration (Figure 4C) were completely reversed by wt-SphK1. Thus, restoring SphK1 expression blocked miR-3677-induced anti-OS-1 cell activity.

We further hypothesized that miR-3677 should be ineffective in SphK1-depeleted cells. Therefore, a CRISPR-Cas9-SphK1-KO-GFP construct was transduced to OS-1 cells. Via GFP-sorting, two stable cell lines, koSphK1-sL1 and koSphK1-sL2, were established (Figure 4D). These cells showed complete SphK1 depletion (Figure 4D). As compared to cells with CRISPR-Cas9 control construct (“Cas9-C”), proliferation (Figure 4E) and migration (Figure 4F) in the koSphK1 OS-1 cells were significantly inhibited. In koSphK1 cells miR-3677 overexpression (by lv-pre-miR-3677) or inhibition (by lv-antagomiR-3677) was unable to alter cell proliferation (Figure 4E) and migration (Figure 4F). Thus, miR-3677 failed to affect cell functions in koSphK1 cells, further confirming that SphK1 is the primary target of miR-3677 in OS cells.

### MiR-3677 is downregulated in human OS tissues

At last we tested the expression of miR-3677 in human tissues. In human OS tissues (“T”, from eight independent primary OS patients [18]), miR-3677 expression was 40% lower than that in the surrounding normal bone tissues (“N”) (Figure 4G). Importantly, miR-3677 reduction in OS tissues was correlated with *SphK1* mRNA elevation (Figure 4H). The latter was over 6-fold higher in OS tissues than that in normal bone tissues (Figure 4H). Therefore, in human OS tissues miR-3677 expression is downregulated, correlating with *SphK1* mRNA upregulation.

## DISCUSSION

Few studies have focused on the function of miR-3677 in human cells. Studies revealed miR-3677 is negatively correlated with overall survival of hepatocellular carcinoma (HCC) patients [19, 20]. Zorniak et al., have shown that the mucosal miR-3677 expression is elevated in patients with cirrhotic gastric antral vascular ectasia (GAVE) [21]. Its potential targets and its expression in human OS are, however, largely unknown.

The results of this study suggest that miR-3677 is a SphK1-targeting miRNA, which inhibits human OS cell progression. RNA pull down assay results demonstrated that biotinylated-miR-3677 directly associated with SphK1 mRNA in OS-1 cells. Forced overexpression of miR-3677 in OS cells inhibited SphK1 3’-UTR activity, causing downregulation of SphK1 mRNA and protein. Contrarily, lv-antagomiR-3677-induced miR-3677 inhibition increased SphK1 3’-UTR activity and expression. Furthermore, SphK1 3’-UTR activity and expression were inhibited only by WT-miR-3677 mimic, but not by the mutant miR-3677 mimics failing to bind SphK1 3’-UTR.

Functional studies revealed that OS cell growth, proliferation and migration were largely inhibited with ectopic miR-3677 overexpression, but augmented with forced miR-3677 inhibition. Furthermore, miR-3677 overexpression induced apoptotic death in OS cells. In human OS tissues, miR-3677 expression was downregulated, correlating with SphK1 mRNA upregulation. These results implied that miR-3677 targets and silences SphK1, efficiently inhibiting human OS cell progression.

Our results further imply that *SphK1* silencing should be the primary reason of miR-3677-induced anti- OS cell activity. First, mimicking lv-pre-miR-3677-induced actions, SphK1 KO (using CRISPR/ Cas9 strategy) inhibited OS cell proliferation and migration. Second, in SphK1 KO OS-1 cells exogenously altering miR-3677 expression, by lv-pre-miR-3677 or lv-antagomiR-3677, failed to change cell functions. Third, lv-pre-miR-3677-induced anti-OS-1 cell activity was reversed by forced expression of an UTR-depleted *SphK1*. Therefore, targeting *SphK1* by miR-3677 induced significant anti-OS cell activity.

OS is a neoplastic growth in bone tissue, with malignant proliferation and metastasis of OS cells [22]. The current anti-OS therapies are very limited. Therefore, exploring novel therapeutic targets and developing alternative treatment strategies are urgently required [22]. The current study demonstrated that targeting *SphK1* by miR-3677 inhibited human OS cell progression. MiR-3677, and possible other SphK1-targeting miRNAs, could be novel therapeutic advance for OS treatment.

## MATERIALS AND METHODS

### Chemicals and reagents

Puromycin and polybrene were provided by Sigma-Aldrich Chemicals (St. Louis, Mo). All the antibodies were obtained from Abcam (Cambridge, MA). RNA reagents and Lipofectamine 2000 were obtained from Thermo-Fisher Invitrogen (Shanghai, China). All sequences and plasmids were provided by Shanghai Genechem Co. (Shanghai, China), unless otherwise mentioned.

### Human tissues

The protocols of this study were approved by the Ethic Committee of Nanjing Medical University, according to Declaration of Helsinki. The human OS tissues and the surrounding normal bone tissues from eight (8) written-informed OS patients, were provided by Dr. Liang at Zhejiang University [18]. Tissues were incubated with the described lysis buffer [18], stored in liquid nitrogen before further biochemical analyses.

### OS cells

U2OS and MG-63 established human OS cells were provided by Dr. Liang at Zhejiang University [18]. The OS cells were subjected to mycoplasma/microbial contamination examination every three months. Short Tandem Repeat (STR) profiling, population doubling time (PDT), and cell morphology were routinely (every 3-4 months) checked to verify their genotypes. Three independent patient-derived primary human OS cells [23], OS-1, OS-2 and OS-3, were provided by Dr. Ji at Nanjing Medical University [23], cultured under described protocols [23, 24]. The primary OS cells at passage 3-10 were utilized.

### qPCR

Total cellular RNA, extracted using the TRIzol reagents, was revere transcripted [25]. Under the ABI Prism 7900 system the quantitative real time-PCR (qPCR) was performed (using described protocols [26, 27]). The melt curve analyses were applied to calculate product melting temperature. *Glyceraldehyde-3-phosphatedehydrogenase* (*GAPDH)* was tested as the reference gene and the internal control, with quantification through the 2^−∆∆*Ct*^ method. MiR-3677 expression was normalized to U6. The primers utilized in this study were listed in Table 1.

**Table 1:** Primers for qPCR assay.

**Genes**	**Forward sequence (5’-3’)**	**Reverse sequence (5’-3’)**
*miR-3677*	CAGTGGCCAGAGCCCTGCA	GAACATGTCTGCGTATCTC
*U6 RNA*	CTCGCTTCGGCAGCACAT	TTTGCGTGTCATCCTTGCG
*SphK1*	GCTGGCAGCTTCCTTGAACCAT	GTGTGCAGAGACAGCAGGTTCA
*GAPDH*	GTCTCCTCTGACTTCAACAGCG	ACCACCCTGTTGCTGTAGCCAA

### Forced miR-3677 overexpression or inhibition

The pre-miR-3677 sequence (GGCAGUGGCCAGA GCCCUGCAGUGCUGGGCAUGGGCUUCUCGUGGGCUCUGGCCACGGCC) and its anti-sense sequence (anti-pre-miR-3677) were synthesized and verified by Shanghai Genechem Co. Each was separately inserted into the GV369 construct (Shanghai Genechem Co.). The construct, together with lentivirus helper plasmids (psPAX2 and pMD2.G [28], from Shanghai Genechem Co.), were co-transfected to HEK-293T cells, generating the pre-miR-3677-expressing lentivirus (“lv-pre-miR-3677”) or the pre-miR-3677 anti-sense lentivirus (“lv-antagomiR-3677”). Virus was enriched, filtered and added to human OS cells (cultured in the polybrene-containing complete medium). Puromycin (5.0 μg/mL) was included to select stable cells, with mature miR-3677 (sequence, CAGUGGCCAGAG CCCUGCAGUG) expression tested by qPCR.

### Transfection of miR mimic

OS cells were seeded into the six-well plates (at 50% confluence), transfected with 500 nM of the applied miR mimic through using a Lipofectamine 2000 protocol [29].

### SphK1 3'-UTR activity assay

Briefly, the human *SphK1* 3’-UTR with the putative binding sites of miR-3677 (position 235-242) was amplified by PCR, then inserted into the firefly luciferase reporter vector, pGL4.13 (luc2/SV40) (Promega) at the XbaI site and downstream from the stop codon of the luciferase gene. The plasmid, along with the Renilla luciferase reporter vector and pRL-SV40 (Promega), were co-transfected to human OS cells by Lipofectamine 2000. Cells were then subjected to applied genetic modifications, with the SphK1 3'-UTR luciferase activity tested through a Promega kit [29].

### RNA-pull down assay

The detailed protocols of RNA-Pull down assay, using the Pierce Magnetic RNA Pull-Down Kit, were described early [30, 31]. OS-1 cells were transfected with biotinylated miR-3677 mimic or control mimic (100 nmol/L) for 36h, and cells were harvested [31]. The lysates were incubated with streptavidin-coated magnetic beads to pull-down biotin-captured RNA complex [30], and the latter was purified by the RNeasy Mini Kit (QIAGEN), with expression of *SphK1 mRNA* tested by qPCR. Its level was always normalized to input controls.

### Western blotting

OS cells, with the applied genetic modifications, were harvested using the described lysis buffer [32]. Twenty μg lysate proteins per sample were separated by 10- 15% SDS gels, and transferred onto PVDF blots (Millipore, Shanghai, China). The blots were blocked, probed with applied primary and second antibodies [32]. The enhanced chemiluminescence (ECL) detection system was applied to visualize the targeted protein bands (based on the molecular weights), using x-ray films. For all the Western blotting assays, each lane was loaded with exact same amount of quantified protein lysates, then the same set of lysate samples were run in parallel (“sister”) gels. The ImageJ software was utilized for data quantification [33, 34].

### EdU staining

OS cell proliferation was tested by the 5-ethynyl-20-deoxyuridine (EdU) Apollo-488 Kit (Ribo-Bio, Guangzhou, China) [33, 34]. OS cells with applied genetic modifications were cultured for 72h, stained with EdU (5 μM for 2h) and DAPI (for 2h). Cell nuclei were visualized under a fluorescent microscope (Leica). At least 600 cell nuclei per preparation in six random views were counted to calculate the EdU ratio (EdU/DAPI×100%).

### Colony formation

OS-1 cells (5 ×10^3^ per well) with the applied genetic modifications were trypsinized and re-suspended in complete medium with agarose (0.25%). Cells were then plated on the top of 10-cm dishes. Medium was renewed every two days. At day-10, the viable cell colonies were counted manually.

### *In vitro* cell migration

OS cells with applied genetic modifications (0.2 × 10^5^ cells of each treatment in 250 μL medium) were plated in the upper chambers (12 μm pore, Corning, New York, NY) [35], with the lower chambers filled with completed medium (with 12% FBS). After 24h, OS cells invading into the lower chambers were fixed, stained and counted. To exclude cell proliferation mitomycin (3.0 μg/mL, Sigma) was added [35].

### Annexin V FACS

OS cells with the applied genetic modifications were stained with Annexin V and propidium iodide (PI), analyzed by fluorescent-activated cell sorting (FACS) on a FACSCalibur machine (BD Biosciences) [35].

### TUNEL staining

OS cells with the applied genetic modifications were tested by a TUNEL (Terminal deoxynucleotidyl transferase dUTP nick end labeling) In Situ Cell Death Detection Kit (Roche, Shanghai, China). At least 600 cell nuclei per preparation in six random views were counted to determine TUNEL ratio (TUNEL/DAPI×100%).

### Ceramide assay

Using the previously-described protocol [36], the cellular ceramide contents in OS cells were analyzed, with the values expressed as fmol by nmol of phospholipid.

### Ectopic overexpression of SphK1

The lentiviral *SphK1* (with no 3’-UTR region) expression GV369 construct was designed, synthesized and sequence-verified by Shanghai Genechem, then transduced to OS-1 cells with lv-pre-miR-3677. Cells were selected by puromycin for two passages, with SphK1 expression confirmed by qPCR and Western blotting.

### CRISPR/Cas9-induced knockout of SphK1

The small guide RNA (sgRNA) against human SphK1 (Target DNA Sequence: ACCGATAAGGAGCTG AAGGC, PAM sequence AGG) was selected from Dr. Zhang’s lab at MIT, inserted into the lentiCRISPR-GFP plasmid (from Dr. Zhang at Soochow University [37]) containing a puromycin selection gene (Addgene) [37]. OS-1 cells were plated into the six-well plates (1×10^5^ cells per well), and transfected with lentiCRISPR SphK1-KO plasmid. Cells were subjected to FACS-mediated GFP sorting. The single stable cells were screened for SphK1 KO by Western blotting/qPCR. Two stable SphK1 KO cell lines were established. Control cells were transfected with the empty vector.

### Statistical analysis

Date were expressed as means ± standard deviation (SD). The statistical differences were analyzed through the one-way analysis of variance (ANOVA) by the Tukey’s post hoc multiple comparison tests (SPSS 21.0, SPSS co. Chicago, CA). Comparisons between two specific groups were performed by the two-tailed Student t tests (Excel 2007, Microsoft). *P* < 0.05 was considered statistically significant.
